# An in-depth analysis of parameter settings and probability distributions of specific ordinal patterns in the Shannon permutation entropy during different states of consciousness in humans

**DOI:** 10.1007/s10877-023-01051-z

**Published:** 2023-07-29

**Authors:** Michelle Franka, Alexander Edthofer, Andreas Körner, Sandra Widmann, Thomas Fenzl, Gerhard Schneider, Matthias Kreuzer

**Affiliations:** 1 Department of Anaesthesiology and Intensive Care Medicine, University Hospital Rechts Der Isar, Technical University of Munich, Munich, Germany; 2 Department Biology, Ludwig-Maximilians University of Munich, LMU Biocenter, Planegg-Martinsried, Munich, Germany; 3 Institute of Analysis and Scientific Computing, TU Wien, Vienna, Austria

**Keywords:** Permutation entropy, Complexity measures, Monitoring, EEG

## Abstract

As electrical activity in the brain has complex and dynamic properties, the complexity measure permutation entropy (PeEn) has proven itself to reliably distinguish consciousness states recorded by the EEG. However, it has been shown that the focus on specific ordinal patterns instead of all of them produced similar results. Moreover, parameter settings influence the resulting PeEn value. We evaluated the impact of the embedding dimension *m* and the length of the EEG segment on the resulting PeEn. Moreover, we analysed the probability distributions of monotonous and non-occurring ordinal patterns in different parameter settings. We based our analyses on simulated data as well as on EEG recordings from volunteers, obtained during stable anaesthesia levels at defined, individualised concentrations. The results of the analysis on the simulated data show a dependence of PeEn on different influencing factors such as window length and embedding dimension. With the EEG data, we demonstrated that the probability P of monotonous patterns performs like PeEn in lower embedding dimension (*m* = *3*, AUC = 0.88, [0.7, 1] in both), whereas the probability *P* of non-occurring patterns outperforms both methods in higher embedding dimensions (*m* = *5*, PeEn: AUC = 0.91, [0.77, 1]; *P*(non-occurring patterns): AUC = 1, [1, 1]). We showed that the accuracy of PeEn in distinguishing consciousness states changes with different parameter settings. Furthermore, we demonstrated that for the purpose of separating wake from anaesthesia EEG solely pieces of information used for PeEn calculation, i.e., the probability of monotonous patterns or the number of non-occurring patterns may be equally functional.

**Supplementary Information:**

The online version contains supplementary material available at 10.1007/s10877-023-01051-z.

## Introduction

Reliable and accurate EEG monitoring helps anaesthesiologists to navigate general anaesthesia and to avoid anaesthetic levels that may lead to unwanted awareness or that can cause an increased risk for a postoperative neurocognitive disorder [1]. In the context of clinical EEG monitoring almost exclusively spectral analytical approaches are applied. The most widely used system, the bispectral index BIS (BIS; Medtronic, Dublin, Ireland) seems to focus on the power in slow and especially in fast frequency bands [2, 3]. The SEDline (Masimo Corporation, Irvine, CA, USA), Narcotrend (Narcotrend-Group, Hannover, Germany) and Conox (Quantium Medical, Fresenius, Bad Homburg, Germany) monitor also incorporate EEG band power in their algorithm to derive the processed EEG index [4–6]. One monitoring system, the entropy module (GE Healthcare, Helsinki, Finland) applies the Shannon Entropy [7] to evaluate the shape of the power spectrum [8]. Since this step occurs in the frequency domain it should not be confused with the time-domain-based permutation entropy (PeEn) [9], the parameter used in this manuscript. The mentioned monitoring systems evaluate anaesthetic-induced changes in the (power) spectrum of the EEG and calculate an index that inversely correlates with the anaesthetic level or the so-called depth of anaesthesia, albeit this term may not accurately reflect the induced effects of an anaesthetic [10]. To calculate these indices, the information regarding the (power) spectrum is derived by a linear transformation, the Fourier transformation, to obtain the trigonometric functions, the (stationary) signal can be described with. Although the EEG is nonstationary, episodes up to ~ 25 s may be considered stationary [11–13]. But as electrical activity in the brain exhibits complex behavior with chaotic, non-linear dynamic properties, interpretation of EEG data with non-linear, entropic analyses could be useful [14, 15]. For scientific purposes, entropy and complexity measures analysing the EEG in the time domain have been successfully applied to perioperatively recorded data. Especially PeEn proved capable in reliably separating EEG from conscious and unconscious states. PeEn codes the time series into order patterns and seems capable of detecting nonlinearities in the signal [16]. It evaluates the probability distribution of ordinal patterns. Studies showed that various versions of the PeEn perform outstandingly in terms of higher coefficient of determination, prediction probability and less baseline variability compared to other current clinical indices for monitoring the anaesthesia induced with GABAergic agents [17]. As the calculation of PeEn depends on parameter settings like order pattern length and the number of data points of the EEG segment to be investigated [17–19], we decided to evaluate the impact of order pattern length and the length of the EEG segment on the resulting PeEn. To get a better understanding of PeEn behavior in general, we also investigated the occurrence probability of strictly monotonous patterns as well as non-occurring patterns and how these probabilities change with anaesthesia. We based our analyses on simulated data as well as on EEG recordings from volunteers.

## Methods

### Permutation entropy

Permutation entropy is a complexity measure for time series, also described as a signals’ distance to white noise [20]. It was introduced by Christoph Bandt and Bernd Pompe in 2002 with the intention to create a more robust and invariant calculation, compared to non-linear monotonous transformations for real-world data like EEG or cardiovascular signals [9].

**Fig. 1:**
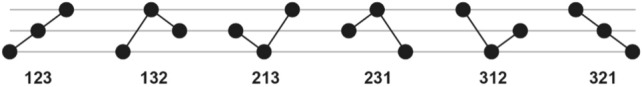
Representation of shapes of possible ordinal patterns for the embedding dimension *m* = *3*

The calculation operates by ranking neighboring values within a pre-defined embedding dimension *m* by position and value, assigning them to a corresponding ordinal pattern. The number of possible ordinal patterns (permutations) depends on the embedding dimension *m*, with *|Ω*_*m*_*|*= *m!,* where *Ω*_*m*_, as the labelling set, contains all these possible ordinal patterns. The rank indices starting from 1, for the smallest value, to *m*, for the highest value. The possible ordinal patterns result from the defined embedding dimension, e.g., for *m* = *3* are *Ω*_*3*_ = *(123,132,213,231,312,321)*. Hence the possible patterns are those shown in Fig. 1.

For demonstration purposes, we chose the time series *X*_*t*_ = *(X*_*1*_*, X*_*2*_*, …, X*_*n*_*)* = *(9, 7, 12, 53, 68, 2)* and the embedding dimension *m* = *3*. First, we rank the first three values (9, 7, 12) and assign them their corresponding ordinal pattern, namely (213). With a time delay of *τ* = *1* we proceed by shifting our embedding dimension one value to the right and start the whole process from the beginning, creating a sequence of ordinal patterns and their occurrence prevalence. The next sequence would then be (7, 12, 53) leading to the rank order pattern of (123) and so on. The last pattern in our example would be (231), derived from the values (53, 68, 2). With this complexity measure method, it is usually assumed that equal values appearing consecutively almost never occur in physiological time series like the EEG, so that we can neglect this scenario [9].

In the following the cardinality of *X*_*t*_ = *(X*_*1*_*, X*_*2*_*,…, X*_*n*_*)* will be the length of *Xt*_*τ*_, thus *n*. In this example, our time series *X*_*t*_ has a total of six values (*n* = *6*). With the determined pattern length of *m* = *3* and a defined time delay of *τ* = *1*, we have four samples of values in total that appear in our time series, in general

1 $$n - \left( {m - 1} \right)\tau$$

The probability *p*_i_ of each ordinal pattern is calculated by its occurrence prevalence, i.e., the total amount of occurrence of the pattern, divided by the number of samples in the examined time series. The set of probability distributions ***P*** of the order patterns then is the foundation for the PeEn which is defined in terms of the Shannon entropy *H*

2 $$H\left( {\varvec{P}} \right) = { }PeEn\left( {m,{\uptau }} \right) = \mathop \sum \limits_{i = 1}^{m!} p_{i } \log \frac{1}{{p_{i} }}$$

with a possible result range of *0* ≤ *H(P)* ≤ *log(m!)*. In our case, the PeEn values were then normalized by the division with the maximum PeEn value for a defined *m*, to get dimensionless quantities in the interval [0, 1]. Large values of PeEn indicate high complexity. The normalization of *H(P)*, according to

3 $$\widehat{PeEn} = \frac{{H\left( {\varvec{P}} \right)}}{{\log \left( {m!} \right)}}$$

leads to *0* ≤ $\widehat{PeEn}$≤*1*, but this normalization, but also the mathematical formulation, rank indices, and terminology may differ from author to author [9, 21]. The notation for *Ω*_*m*_ that is used in this paper is only possible until a *m* of 9, as otherwise the numbers cannot be clearly distinguished from each other.

### Utilised signals

#### Simulated signals

**Fig. 2:**
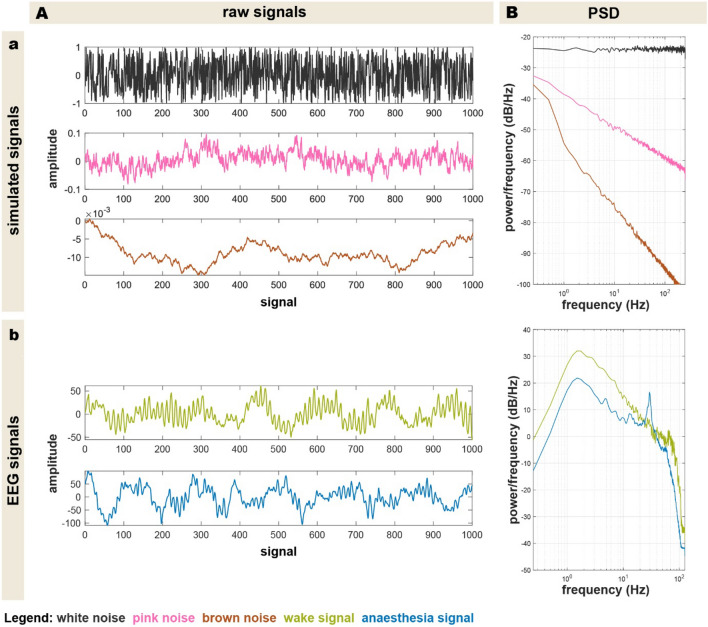
Raw simulated noise signals (white, pink, and brown noise) (Aa) and their subsequent power density spectrum (Ba). While white noise has a flat spectral density, the power density spectrum of pink noise exhibits a 1/f decline, leading to a decrease of power in higher frequency bands. Brown noise follows this trend even more, with a 1/f^2^ decrease of the spectral power. The raw traces of the simulated signals (Aa) depict the degree of randomness well: White noise being an uncorrelated signal, depicting no structure whatsoever, whereas pink and brown noise signals seem to be more rhythmic signals, as they are correlated signals. The raw traces of the human EEG signals are presented in (Ab). The power density spectra of both clinical signals (Bb) depict a similar trend to the respective correlated simulated signals

To perform a precise analysis of PeEn, i.e., to investigate specific pattern occurrences as well as the influence of various parameters, simulated noise signals that resemble EEG features in different states of consciousness were first used [22]. Using simulated data, probable variations between individuals or different consciousness states in real-world data, as well as for instance artefact-related EEG fluctuations can be excluded. White, pink, and brown noise signals were chosen for the analysis. Those signals have different properties, deriving from their respective spectral density 1/f^ß^. White noise signals (ß = 0) depict a maximal random state, which represents all frequencies equally in the power density spectrum (Fig. 2Ba). The signal has no order to it, resembling an uncorrelated random signal. Such a signal does not represent a brain signal in a specific consciousness state [23]. On the other hand, correlated random signals, such as pink or brown noise very well resemble signals of different consciousness states of the brain [24]. The spectral density of pink noise signals follows a 1/f tenor (ß = 1), meaning, the power decreases the higher the frequencies get (Fig. 2Ba). It simulates an EEG signal of a wake mammalian the most. Brown noise, however, approximates an EEG signal of an anaesthetised brain, with even less power in the spectral density of higher frequencies (1/f^2^) compared to the pink signal. Exemplary raw traces of these simulated EEG traces as well as their power density spectra are presented in supplemental Fig. 2a.

#### Volunteer EEG

To evaluate the performance of the single variables to detect different levels of anaesthesia, we used the EEG data from a previously published data set [25]. The study design with stable anaesthesia levels at defined, individualised concentrations allowed for assessment of the performance of these variables. We used 120 s of EEG with a sample rate of 100 Hz extracted during the wake state, a deeper level of (non-burst suppression) general anaesthesia (inter2 in the original publication) and a lighter level of general anaesthesia (inter1 in the original publication) achieved through mono anaesthesia with sevoflurane or propofol. Additionally, we extracted 20 s segments of each of these signals and repeated the statistical analysis with those shorter and more clinically relevant timeframes. Before calculating the parameters, we bandpass filtered the EEG to the 0.5 Hz to 30 Hz range. We chose this range because of the known EMG contamination of the frontal EEG. Of course, the frequency spectrum of the EMG is not limited to frequencies above 30 Hz but it seems to increasingly contaminate the EEG in the higher frequencies [26]. The entropy module’s processed EEG parameter reflecting the hypnotic component of anaesthesia, the state entropy, only considers frequencies up to 32 Hz [27]. The BIS, considering frequencies up to 47 Hz seems strongly influenced by the EMG [28]. Although the cutoff of the low pass is in accordance with the Shannon-Nyquist theorem, the low sample rate may cause a reduction in information content since higher oversampling factors lead to a better resolution. We conducted an analysis of the noise signals that were low-pass filtered at 30 Hz and then down sampled with different factors to show that the effect on PeEn was low. The exclusion of the higher EEG frequencies can help to reduce the influence of muscle activity on the recorded signal [29]. If included like in the case of the bispectral index, wakefulness in the fully paralyzed patient may not be detected [28, 30]. Exemplary raw traces of volunteer EEG traces are presented in Fig. 2Ab, as well as their power density spectrum in Fig. 2Bb.

To assess the difference in the EEG between lighter and deeper anaesthetic levels using a clinically established parameter, we calculated the spectral edge frequency 95 (SEF95). It was obtained from the power spectral density (PSD) of the EEG signals with a frequency resolution of 0.39 Hz (NFFT = 256, sample rate = 100 Hz). The SEF95 was calculated as the frequency, below which 95% of the total power of the EEG signal in the frequency range 0.5–30 Hz were located. To investigate the effect of different sampling rates on PeEn values, we increased the sampling rate of the 20 s EEG segments from 100 Hz to higher sampling rates up to 300 Hz by spline interpolation. To investigate the impact of signal length on PeEn we reduced the 20 s EEG segments in a stepwise approach by 1 s per step. Therefore 0.5 s of EEG were removed from the start and end of the EEG episode until the shortest segments‘ length was 1 s (= 100 data points).

### Parameter settings and calculation

To analyze the influence of different parameters on the PeEn calculation for the simulated signals, window length as well as the embedding dimension were varied. For order pattern generation, a time delay of *τ* = *1* was continuously used, because *τ* > *1* may lead to unintended signal distortions [21]. To evaluate PeEn performance, we used 10 signals of each noise and window lengths of 500, 1000, 2000, 5000, 10,000, 20,000 and 50,0000 data points. The number of data points, i.e., the length of the episodes were arbitrarily chosen, but in a manner to reflect short and long EEG segments. In case of dynamic state transitions the analysed EEG episode should be short to capture the temporal change in the EEG. A parameter like for instance PeEn calculated over longer episodes could help to provide a reliable assessment during steady state levels during anaesthesia maintenance. The PeEn of all these signals was then calculated for an embedding dimension of *m* = *3, 4, 5, 6* and *7*. We also calculated the proportion of strictly monotonous patterns

$$x_{\tau 1} < \, x_{\tau 2} < \, \ldots \, < \, x_{\tau m} or \, x_{\tau 1} > \, x_{\tau 2} > \, \ldots \, > \, x_{\tau m}$$

and of non-occurring patterns, where *p*_*i*_ = *0*.

### Analytical approach

The MATLAB (The Mathworks, Inc., Natick, MA, USA) code (adapted to our needs from Dimitriadis Stravros, 2010) calculates the PeEn, the normalised PeEn and creates a list of the respective probability distributions of all ordinal patterns within the previously defined embedding dimension for the segment. To investigate the occurrence of monotonous patterns, the probabilities of the respective patterns were added and displayed. The probability was calculated by the occurrence prevalence of monotonous patterns divided by the number of samples of values the segment contains. For the analysis of the non-occurring patterns, all ordinal patterns with a probability distribution of 0 were added up. The sum was then divided by the number of all possible ordinal patterns for the respective embedding dimension, which is *m!*.

### Statistical analysis

To compare the performance of distinguishing between different states of consciousness, an area under the receiver operating characteristic curve (AUC) of the occurrence probability of monotonous patterns, as well as the probability of non-occurring patterns and the conventional PeEn was performed. Wake (14 signals) and two different stages of anaesthesia (inter1 and inter2, 15 signals each) from the volunteer EEG data were used [25]. The AUC for dichotomous data is equivalent to the prediction probability [31], which was regularly used to evaluate the performance of commercial processed EEG indices or entropic parameters [32, 33] to distinguish different levels of anaesthesia. The AUC value represents the discriminatory capacity between two classes 0 and 1, with a value of 0.5 meaning no separability of any kind. In this work, the classes represent the consciousness states wake and inter1, as well as inter1 and inter2. We executed this analysis with the whole 120 s segments and then with 20 s signals, the latter extracted from the data points in the middle of the 120 s segments. For the analysis, we used the MES-toolbox for MATLAB [34]. The establishment of the corresponding 95% confidence intervals was obtained by the bootstrap method (10,000 repeats). If the 95% confidence interval does not contain the value indicative of no effect, i.e., AUC = 0.5, the result can be considered significant on a p < 0.05 level [34]. We also investigated the influence of the sample rate on the PeEn by calculating a linear model using the MATLAB *fitlm* function. To evaluate the change in heterogeneity of PeEn for one condition, we calculated the quartile coefficient of variation (CV_Q_), which can be calculated as ${CV}_{Q}(x)=\frac{{Q}_{3}(x)-{Q}_{1}(x)}{{Q}_{3}(x)+{Q}_{1}(x)}$, where Q1 and Q3 are the first and third quartile of the PeEn [35].

## Results

### Simulated data

#### Influence of embedding dimension and pattern length on PeEn

**Fig. 3:**
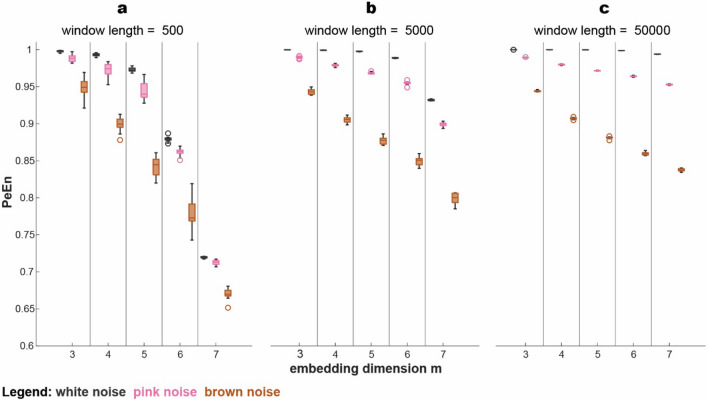
Changes in PeEn with increasing embedding dimension for window lengths of 500, 5000 and 50,000 data points. White noise signals have the highest PeEn values compared to pink and brown noise signals in any given setting. PeEn generally decreases with increasing *m*, especially for short window lengths. An increasing window length led to increased PeEn values in higher embedding dimensions in all three simulated signals. The variability of PeEn within each of the simulated signals decreases with higher window lengths and depends on the properties of the signal. In the boxplots, one whisker connects the upper quartile to the nonoutlier maximum (the maximum data value that is not an outlier), and the other connects the lower quartile to the nonoutlier minimum (the minimum data value that is not an outlier). Outliers are values that are more than 1.5 · interquartile range away from the top or bottom of the box as defined as default setting in the MATLAB boxchart function

For our tested settings we found the expected order of PeEn values. White noise had the highest PeEn, followed by pink noise and brown noise having the lowest PeEn. For segments of 500 data points the absolute PeEn values of each simulated signal decreased with higher *m*. This trend continued throughout all tested window lengths but got far more subtle with increasing number of data points, especially in higher embedding dimensions. Consequently, it seemed that the higher the embedding dimension, the more influence the window length had. Moreover, for a window length of 500 and up to an embedding dimension of *m* = *5* the differences in PeEn between white, pink and brown noise increased but then decreased again for the two highest embedding dimensions (*m* = *6, m* = *7*) (Fig. 3a). For window lengths of 5000 and especially 50,000 data points, however, the differences in PeEn values between the three simulated signals kept increasing with higher embedding dimension resulting in the biggest difference between the three signals at settings of window length = 50,000 and *m* = *7* (Fig. 3b and c). The variability in PeEn values for each of the ten signals of one parameter setting and color depended on two components. Firstly, the signal properties itself, with white noise having the lowest variability compared to the colored signals. Secondly, the number of data points was also an influencing factor as less variability in PeEn values occurred in the signals with greater window lengths (Fig. 3). This change is also reflected in the CV_Q_ being lower for colored noise and for higher window lengths as presented in supplemental Figure S1.

#### Monotonous and non-occurring patterns

**Fig. 4:**
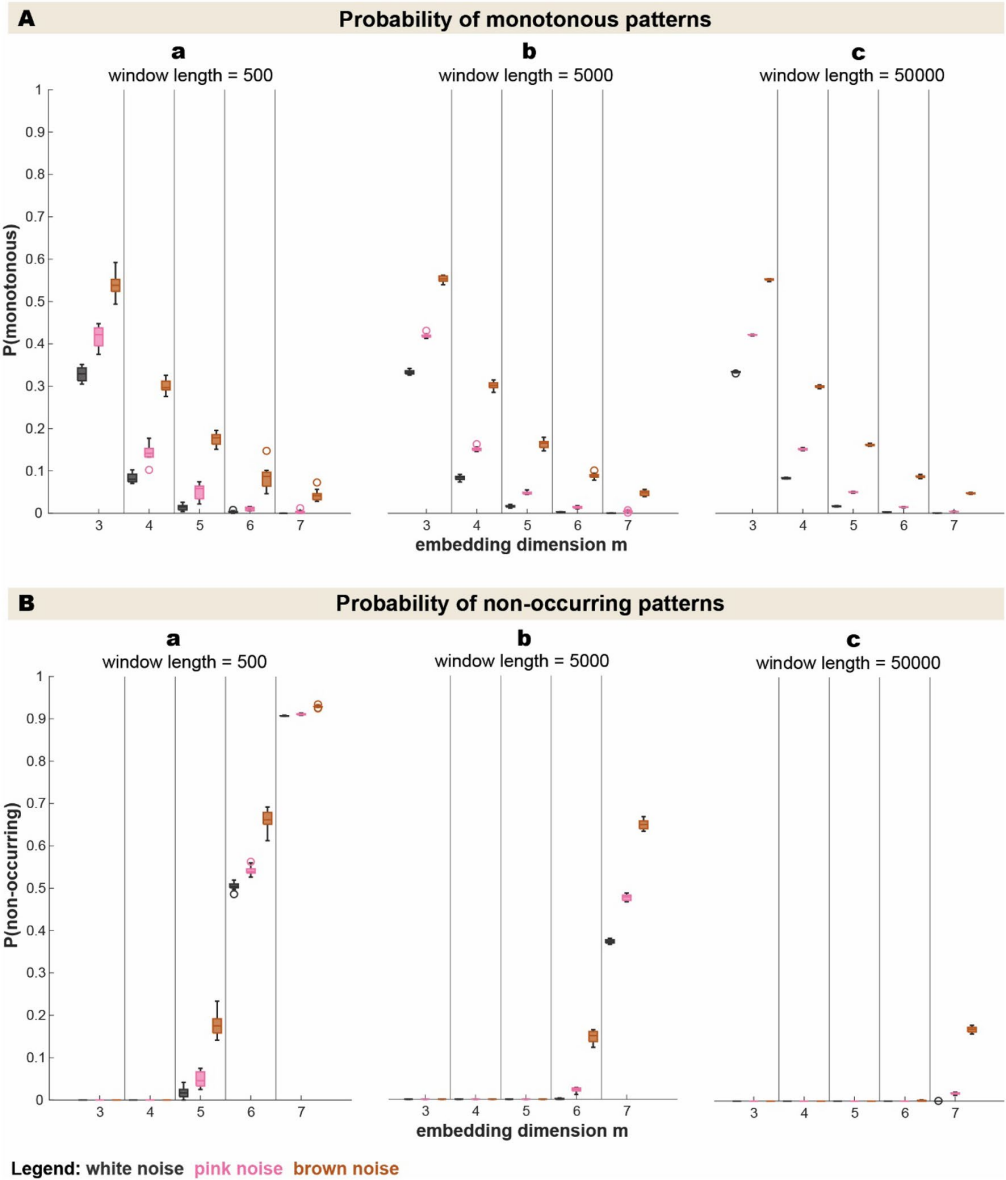
Probability of monotonous and non-occurring patterns for various embedding dimensions and window lengths. With increasing embedding dimensions, the probability of monotonous patterns decreases across all window lengths (4A). All ordinal patterns occur in embedding dimensions *m* = *3* and *m* = *4* but with higher embedding dimensions, the probability of non-occurring patterns increases (4B). Window length, however, weakens this trend. Monotonous patterns as well as non-occurring patterns are most prominent in brown noise, followed by pink noise and lastly by white noise. In the boxplots, one whisker connects the upper quartile to the nonoutlier maximum (the maximum data value that is not an outlier), and the other connects the lower quartile to the nonoutlier minimum (the minimum data value that is not an outlier). Outliers are values that are more than 1.5 · interquartile range away from the top or bottom of the box as defined as default setting in the MATLAB boxchart function

With a closer look on the proportion of monotonous patterns and non-occurring patterns, the proportion of monotonous patterns decreased with increasing *m* while the number of non-occurring patterns increased as shown in Fig. 4. All ordinal patterns did occur in embedding dimension *m* = *3* and *m* = *4* in all examined window lengths. Although the likelihood of patterns not occurring increased with *m*, an increasing window length, however, reduced the probability of non-occurring patterns. This can be observed by comparing Fig. 4Ba with Bc: With 500 data points, patterns start to not occur from an embedding dimension of *m* = *5* on, in the 50,000 data point window, this does not start up until an embedding dimension of *m* = *7*. Brown noise always had the highest proportion of monotonous and non-occurring patterns, followed by pink and then white noise. Again, the variability decreased with increasing window length and depends on properties of the signal with brown noise having the highest variability.

### Volunteer EEG

**Fig. 5:**
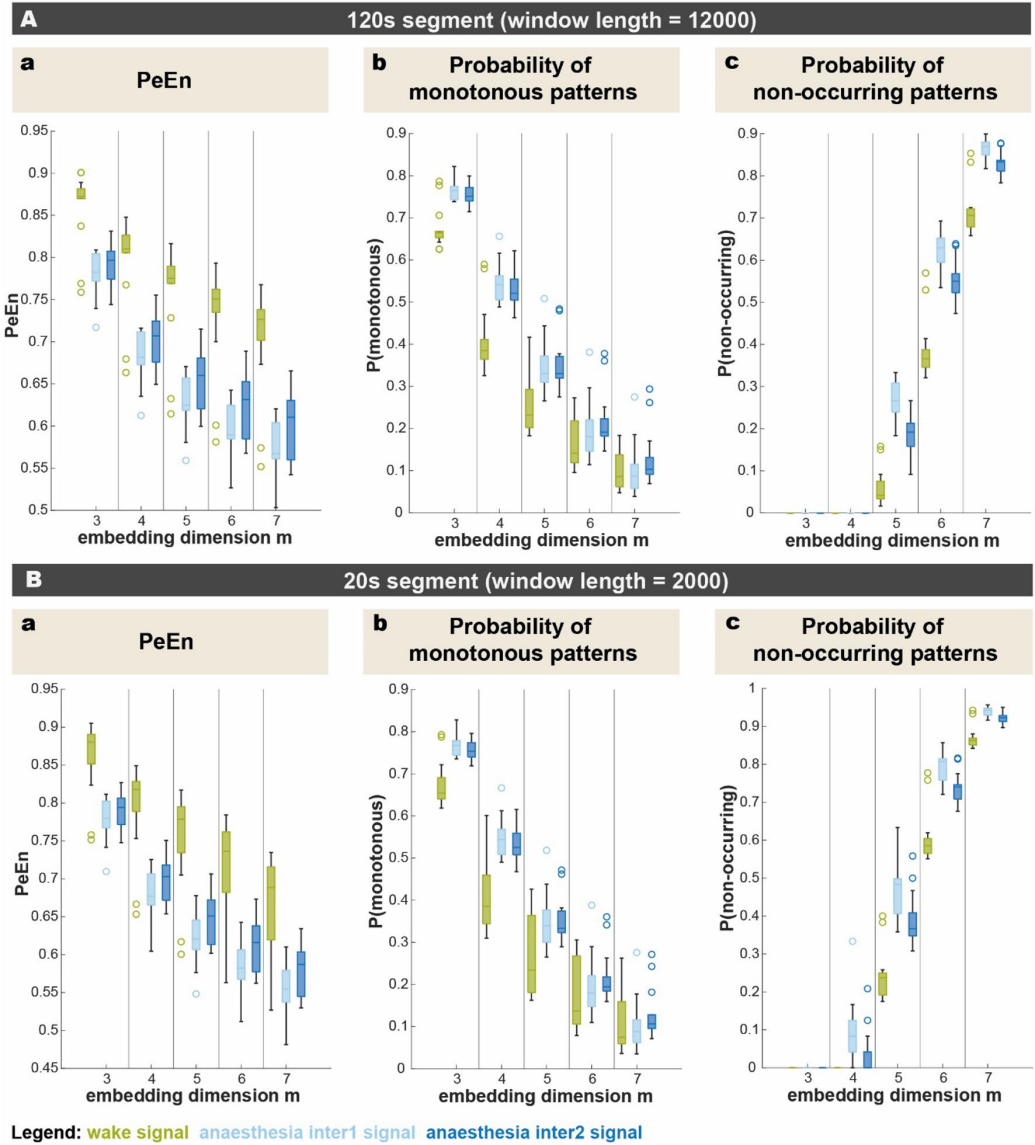
Analysis of PeEn, probability of monotonous and non-occurring patterns in clinical EEG signals of 120 s and 20 s segments. In both segment lengths, the PeEn as well as the probability of monotonous patterns decreases with higher embedding dimension, with wake signals generally having higher values than the two anaesthesia signals. As for the probability of non-occurring pattern an increasing embedding dimension causes a higher proportion of non-occurring patterns. Here, wake signals generally have the lowest values, compared to the anaesthesia signals. The comparison of the two anaesthesia signals shows slightly higher PeEn values and less non-occurring patterns for the deeper-level anaesthesia signals (inter2), compared to the lighter-level ones (inter1). For the most part, the 120 s and the 20 s segments do not differ much from each other, except for the fact, that the variability in values is higher in the shorter segment, compared to the longer ones and that the start of patterns not occurring is at an embedding dimension of *m* = *5* for the longer and at *m* = *4* for the shorter segments. In the boxplots, one whisker connects the upper quartile to the nonoutlier maximum (the maximum data value that is not an outlier), and the other connects the lower quartile to the nonoutlier minimum (the minimum data value that is not an outlier). Outliers are values that are more than 1.5 interquartile range away from the top or bottom of the box as defined as default setting in the MATLAB boxchart function

In both, the analysis of the influence of different parameters as well as the analysis of probability distributions of monotonous and non-occurring patterns, the results of the wake versus the two anaesthesia EEG signals resembled the ones of their corresponding simulated signals (pink versus brown noise signals). For the most part, wake signals had a higher PeEn value than the two anaesthesia signals. With higher embedding dimensions, PeEn and the probability of monotonous patterns decreased. For the 120 s segment, again, in an embedding dimension of *m* = *3* and *m* = *4* all ordinal patterns did occur, but as *m* increased, the probability of non-occurring patterns also increased. This is also true for the 20 s segment, except for patterns already not occurring in an embedding dimension of *m* = *4*. Apart from this, the probability of non-occurring patterns is higher for the anaesthesia segments, compared to the wake signals. Contrary to our intuition, the comparison of the two anaesthesia signals shows slightly higher PeEn values, as well as less non-occurring patterns at any given setting for the deeper-level anaesthesia signals (inter2), compared to the lighter-level ones (inter1). The differences between the 120 s and the 20 s segments related mainly to the differences in variability across window lengths observed in the analysis of the simulated signals above: The variability of PeEn values of the signals at a given setting was higher in shorter signal segments compared to the longer ones (Fig. 5).

### Wake vs. (“light”) anaesthesia (Inter1)

In distinguishing different consciousness states from each other, PeEn values performed slightly better with increasing embedding dimension with AUC values ranging from 0.88 [0.70; 1] (*m* = *3*) to 0.91 [0.77; 1] (*m* = *7*) in the 120 s segment. A similar trend could be observed in the 20 s segment. The probability of monotonous patterns performs similar to the conventional PeEn calculation up to an embedding dimension of *m* = *5*. After that, its distinguishing performance decreased and was not significant anymore. Using the probability of non-occurring patterns for distinguishing different states of consciousness, only higher embedding dimensions, starting from *m* = *5* in the 120 s and *m* = *4* in the 20 s segment showed significant results. However, those results had the highest discriminatory capacity compared to the PeEn and the monotonous patterns with AUC values up to 1 [1, 1] (*m* = *5*).

#### “Deeper” vs. “lighter” anaesthesia (Inter2 vs. Inter1)

The EEGs of the lighter level of anaesthesia showed significantly (p = 0.011) higher SEF values (11.7 Hz [10.9, 13.2] Hz) compared to the deeper anaesthesia levels (11.0 Hz [8.5, 11.6] Hz). The AUC indicated a relevant effect of the anaesthetic level on SEF values with AUC = 0.76 [CI: 0.57–0.9]. The corresponding boxplots are presented in supplemental Figure S2.

PeEn as well as the probability of monotonous patterns produced no significant results thus had no discriminatory capacity in distinguishing between the two anaesthesia levels, neither in the 120 s, nor in the 20 s segment. The probability of the non-occurring patterns, however, did very well distinguish between those states from an embedding dimension of *m* = *5* in both segment lengths (Table 1).

**Table 1:**
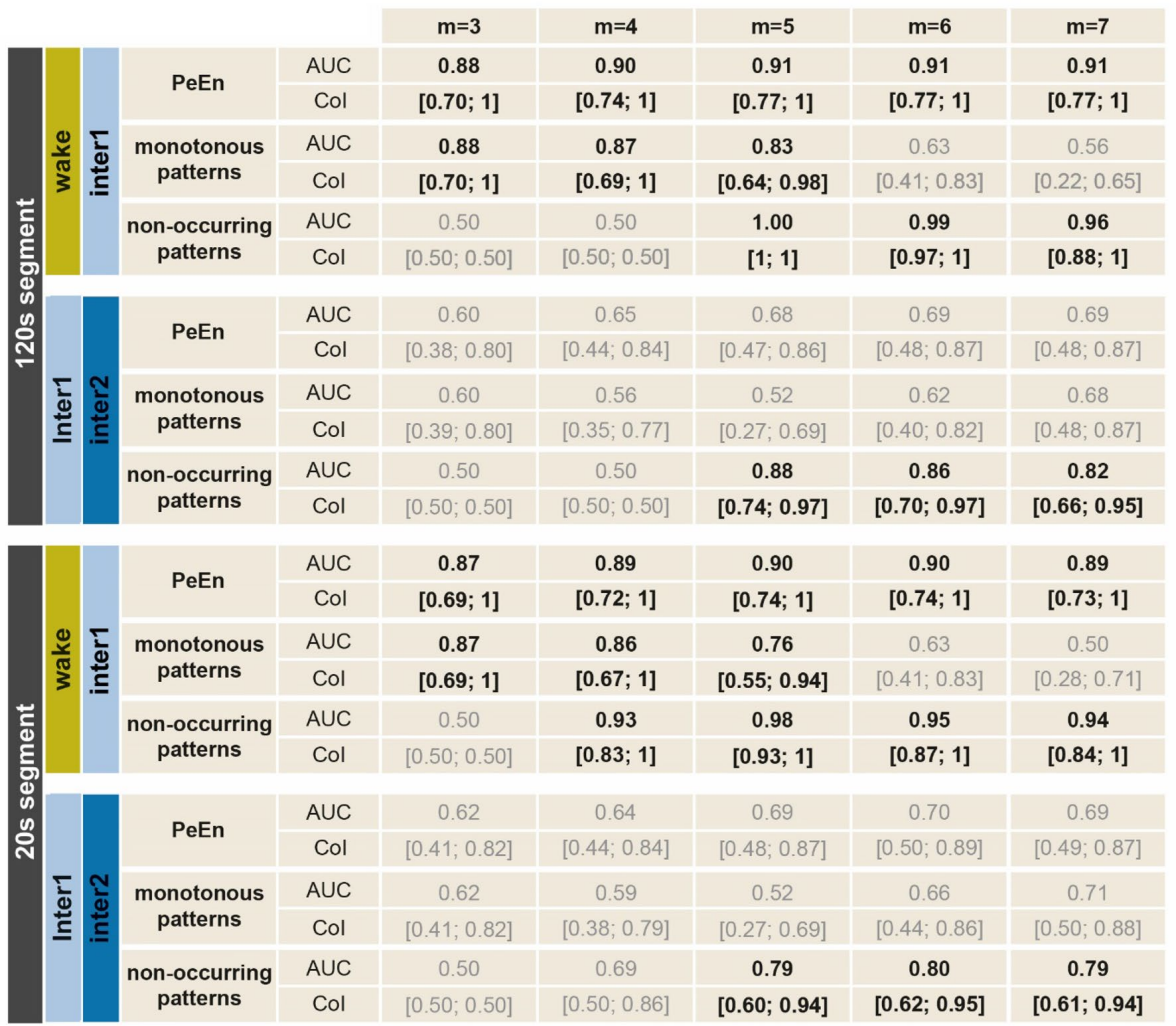
Area under the curve analysis (AUC) results for wake vs. anaesthesia (inter1) and deeper vs. lighter anaesthesia levels (inter2 vs. inter1) for 120 s and 20 s segments with respective confidence intervals (CoI). Bold cells indicate a significant difference based on a 95% CoI exclusive 0.5

#### Influence of sample rate and EEG segment length

The interpolation of the signals, i.e., an increasing sample rate, significantly decreased the PeEn values (p < 0.001) for all states. Linear Regression showed a decrease of the PeEn values by 0.12 or 0.10 per 100 Hz increase in sampling rate, respectively (Wake: PeEn = 0.97–1.2e^−3^*sampling rate, R-squared = 0.96; inter1: PeEn = 0.85–1.0e^−3^*sampling rate, R-squared = 0.94; inter2: PeEn = 0.87–1.0e^−3^*sampling rate, R-squared = 0.95). The performance, i.e., the AUC in separating the different states was not influenced with AUC remaining in the 0.87–0.88 for PeEn and monotonous patterns range to separate wake and the light level of anaesthesia and it was 0.62–0.64 for the separation between light and deep levels of anaesthesia. In the supplement, we present the corresponding Table S1 and Figure S4. The EEG segment length did not influence the PeEn (m = 3) and the percentage of monotonous patterns as well. There were no non-occurring patterns in any of the short segments. The corresponding plots are presented in supplemental Figure S5 and supplemental Table S2 contains the statistical information. To evaluate the impact of down sampling of a low pass filtered signal, we used the different types of noise. Supplemental Figure S6 and supplemental Table S3 show that the sampling rate did not change the AUC of the PeEn, except for the sampling rate of 64 Hz. This may be caused by aliasing due to a violation of the Shannon-Nyquist theorem, because filters are not ideal.

## Discussion

### Analysis of simulated signals

Our results show a dependence of PeEn on different influencing factors such as episode length, i.e., the number of data points, or the embedding dimension that defines the number of possible ordinal patterns. In order to systematically investigate these influences, we used simulated noise signals as well as clinical data. The noise signals, except the white noise, share spectral features that correlate with the spectral EEG features at different states [22]. Pink noise is characterised by a 1/f decrease of the spectral power in the log–log representation and is a very common feature in biological systems [36]. Regarding the EEG it seems to resemble the power with frequency decrease during relaxed awake states [22]. Brown noise, i.e. 1/f^2^, shows a steeper decrease in the spectrum with frequency. It may share the slope with the EEG during unconscious states like general anaesthesia [22]. Therefore, the (permutation) entropy will be higher for pink than for brown noise, because of a less uniform probability distribution. For white noise, PeEn will be maximal [20].

Our results show these differences in PeEn between the three types of noise. Per definition the maximum PeEn is *log(m!)*. Hence, for a given EEG or noise time series, PeEn will increase with *m*. However, when looking at the normalised PeEn we could observe a decrease in values with increasing *m* which can be explained by an increasing number of *non-occurring* patterns. This decrease is steeper for smaller window lengths. For segments with less or close the amount of sample of values than possible ordinal patterns (*m!*), the probability of each ordinal pattern to appear was lower compared to longer time series, where the probability increased again, as non-occurring patterns inversely correlate with the number of data points. For example a time series of 1000 data points and an embedding dimension with *m* = *6* (i.e. *6!* = *720* possible patterns) will lead to a high number of non-occurring patterns. This also explains why the gap between the colored noise signals increases with greater window length.

The decrease in the *probability of strictly monotonous patterns* can be explained by the higher number of possible ordinal patterns (occurring and non-occurring), resulting from higher embedding dimensions. The occurrence prevalence in brown noise (followed by pink and then by white noise) is due to the properties of the signal: slow, rhythmic oscillations will lead to a higher occurrence in strictly rising or falling monotonous patterns.

Questioning the PeEn to be a measure for EEG complexity, Berger et al. showed that the PeEn decrease can be explained by less zero-crossings. Evaluating the zero-crossings was described for EEG analysis almost 60 years ago [37]. One drawback of PeEn for the purpose of anaesthesia monitoring may be a rather poor performance of separating different levels of non-burst suppression anaesthesia [38]. But regardless of PeEn reflecting complexity, we could observe an increase in the probability of strictly monotonous patterns from white to pink to brown noise as well as an increase of non-occurring patterns for higher *m*.

### Analysis of EEG data

In the context of separating wake from anaesthesia EEG, the performance of PeEn remained stable, and if anything, slightly increased with higher *m*. Since there are *m!* possible order patterns the number of non-occurring patterns increases with *m*. Using the monotonous patterns, a similar discriminatory capacity in embedding dimensions up to *m* = *5* can be found. However, in higher embedding dimensions the number of non-occurring patterns seems to function as an even better separator of these states. Mechanistically the explanation seems straightforward: During wakefulness the EEG can be characterised by fast oscillatory activity with low amplitudes and the activity shifts to a slower rhythm with higher amplitudes during general anaesthesia [24]. Even if only lower frequencies are considered for analysis, the EEG slows during anaesthesia with GABAergic drugs and the amplitudes increase. Before processing, the EEG is usually low pass filtered to prevent a dominant influence of distortions such as muscle activity which for instance can increasingly contaminate the recorded signal with increasing frequencies [39]. In our case, we only included oscillatory activity up to 30 Hz, dismissing higher frequencies that also carry important EEG information during the wake state. Still, with these filter settings the wake EEG is faster and of lower amplitude than the anaesthesia EEG. Under anaesthesia, with a very rhythmic signal, the number of longer (e.g. *m* ≥ *5*) patterns that really occur will be limited and hence the number of non-occurring patterns will increase. But because of the 1/f characteristics in the EEG, there will always be slow activity in the signal that will also lead to monotonous patterns or patterns with only one or two peaks. Hence the number of non-occurring patterns in the awake EEG will be lower.

Examining the discrimination capacity between the *two anaesthesia levels*, counterintuitively, the PeEn values were lower for the “lighter” level of general anaesthesia (inter1), than for the “deeper” level of general anaesthesia (inter2). This was due to the spectral features of the EEG at the different levels. The SEF analysis revealed significantly lower SEF for the deeper level, fully in accordance with presented results [40]). As can be observed in supplemental Figure S3, the EEG during inter1 had significantly higher alpha-band power leading to a lower PeEn. We could show previously that an increase in alpha power causes a decrease in PeEn [41]. Moreover, regardless of the level of anaesthesia, detecting that there actually are two anaesthesia states (inter1 versus inter2) PeEn as well as the probability of monotonous patterns did not produce significant results. This was rather expected, as this was also the case in the original publication from Horn et al., where it was explained with PeEn being more a separator for wake vs. anaesthetised than for different anaesthesia levels [25]. However, the non-occurring patterns performed exceptionally well and produced significant results in embedding dimensions equal or greater than *m* ≥ *5*. It seems that with a high number of ordinal patterns, the differences between similar consciousness states are still high enough in terms of the number of non-occurring patters, to distinguish between them. This shows that the information content of the probability distribution of all ordinal patterns calculated by the PeEn reflects a specific consciousness state worse than the much simpler approach of the sheer counting of non-occurring patterns.

Focusing on the results for an embedding dimension of *m* = *3*, the analysis of our EEG data showed that the performance to distinguish the awake state from an anaesthetic level without burst suppression with PeEn was good. Although an increase in sample rate caused PeEn to decrease and the percentage of monotonous patterns to increase, the performance of these parameters was not affected. The change in the parameter values is based on the creation of additional monotonous patterns, due to the introduction of more sample points by the interpolation. Also, the EEG segment length did not influence the parameters, indicating that PeEn can also be used for very short segments. Using the probability of strictly monotonous patterns led to a comparable performance with only using a part of the information used for PeEn calculation. Berger et al. described that the PeEn may in certain cases be substituted by the entropy of peaks that can be derived by generating the second derivative of the time series [21]. We could show that the probability of monotonous patterns seems to also function as a separator between wake and anaesthesia EEG. As for peak detection it may be sufficient to perform a differentiation and hence create a vector from the time series that only consists of “ + ” and “−”. Hence, the step of generating order patterns is not necessary. The entropy of difference that also seems applicable to EEG and other neurophysiological data follows the approach of evaluating the probabilities of “ + ” and “−” patterns and therefore does not require order pattern generation [42, 43].

### Implications for EEG-based monitoring

PeEn seems to be equivalent or even better in its performance to separate wake EEG from anaesthesia EEG compared to spectral methods [38]. But if PeEn really reflects the complexity of the signal or its information content is less clear. At least for *m* = *3* the PeEn seems to be an estimate of the centroid of the weighted power spectrum [34]. For higher *m* such a relation has not been yet described, but with our work we can show that for the purpose of separating wake from anaesthesia EEG pieces of information used for PeEn calculation, i.e., the probability of monotonous patterns or the number of non-occurring patterns may be equally functional.

### Limitations

The purpose of this article is to introduce the analysis of monotonous pattern and of the number of non-occurring patterns. Therefore, an analytical focus was on simulated signals that resemble EEG features as recorded during wakefulness or levels of anaesthesia. The analysis of the EEG was limited to stable episodes of wake and anaesthesia EEG where anaesthesia was maintained with propofol or sevoflurane. So, we cannot make any claims for other anaesthetics as their EEG characteristics may be very different [34]. Also, in terms of opioids etc. we cannot draw any conclusions. For order pattern generation we used a *τ* = *1*. Numerous publications used other time lags as well to calculate PeEn. The use of higher lags of *τ* ≥ *2* can lead to discontinuous frequency dependencies [44, 45], and hence we decided to not include higher lags since that may overcomplicate the article. For the EEG analyses, we used a rather old data set, but since this data was recorded during stable levels of general anaesthesia, it was perfect for our approach. Also, we only used EEG information after a 30 Hz low pass filtering. This approach may not consider useful information from higher frequencies. But because of the known interference of muscle activity with the EEG, especially in awake patients and the known low index values [28, 30] when relying on higher frequencies for the fully paralyzed and awake patient, we feel that the focus on the lower frequencies makes sense. Also the chosen sampling frequency of 100 Hz was low and higher sampling rates would help to contain more information in the signal. Further, low sample rates may influence the nonlinear analysis of time series [46]. Hence future investigations should consider EEG data with higher sample rates. We also did not evaluate the performance for the used substances individually. This should be done in the future in a larger patient cohort.

### Supplementary Information

Below is the link to the electronic supplementary material.

Supplementary file1 (DOCX 4038 kb)

## References

[1] Chen X (2022). A randomized trial: bispectral-guided anesthesia decreases incidence of delayed neurocognitive recovery and postoperative neurocognitive disorder but not postoperative delirium. Am J Transl Res.

[2] Rampil IJ (1998). A primer for EEG signal processing in anesthesia. Anesthesiology.

[3] Connor CW (2022). Open reimplementation of the BIS algorithms for depth of anesthesia. Anesth Analg.

[4] Kreuer S, Wilhelm W (2006). The Narcotrend monitor. Best Pract Res Clin Anaesthesiol.

[5] Drover D, Ortega HR (2006). Patient state index. Best Pract Res Clin Anaesthesiol.

[6] Jensen E (2014). Monitoring hypnotic effect and nociception with two EEG-derived indices, qCON and qNOX, during general anaesthesia. Acta Anaesthesiol Scand.

[7] Shannon C (1948). A mathematical theory of communication. Bell System Techn J.

[8] Viertio-Oja H (2004). Description of the entropytm algorithm as applied in the Datex-Ohmeda S/5 tm entropy module. Acta Anaesthesiol Scand.

[9] Bandt C, Pompe B (2002). Permutation entropy: a natural complexity measure for time series. Phys Rev Lett.

[10] Sleigh JW (2011). Depth of anesthesia: perhaps the patient isn't a submarine. Anesthesiology.

[11] Cohen BA, Sances A (1977). Stationarity of the human electroencephalogram. Med Biol Eng Comput.

[12] Kreuzer M (2014). Non-stationarity of EEG during wakefulness and anaesthesia: advantages of EEG permutation entropy monitoring. J Clin Monit Comput.

[13] Kawabata N (1976). Test of statistical stability of the electroencephalogram. Biol Cybern.

[14] Elbert T (1994). Chaos and physiology: deterministic chaos in excitable cell assemblies. Physiol Rev.

[15] Pritchard WS, Duke DW, Krieble KK (1995). Dimensional analysis of resting human EEG II: surrogate-data testing indicates nonlinearity but not low-dimensional chaos. Psychophysiology.

[16] Zunino L, Kulp CW (2017). Detecting nonlinearity in short and noisy time series using the permutation entropy. Phys Lett A.

[17] Liang Z (2015). EEG entropy measures in anesthesia. Front Comput Neurosci.

[18] Jordan D (2008). Electroencephalographic order pattern analysis for the separation of consciousness and unconsciousness: an analysis of approximate entropy, permutation entropy, recurrence rate, and phase coupling of order recurrence plots. Anesthesiology.

[19] Olofsen E, Sleigh JW, Dahan A (2008). Permutation entropy of the electroencephalogram: a measure of anaesthetic drug effect. Br J Anaesth.

[20] Bandt C (2017). A New Kind of Permutation Entropy Used to Classify Sleep Stages from Invisible EEG Microstructure. Entropy.

[21] Berger S (2017). Permutation entropy: too complex a measure for EEG time series?. Entropy.

[22] Colombo MA (2019). The spectral exponent of the resting EEG indexes the presence of consciousness during unresponsiveness induced by propofol, xenon, and ketamine. Neuroimage.

[23] Brown EN, Lydic R, Schiff ND (2010). General anesthesia, sleep, and coma. N Engl J Med.

[24] Ying Jiang C-KP, Yuesheng Xu (2011). Hierarchical entropy analysis for biological signals. J Comput Appl Math.

[25] Horn B (2009). A combination of electroencephalogram and auditory evoked potentials separates different levels of anesthesia in volunteers. Anesth Analg.

[26] Whitham EM (2007). Scalp electrical recording during paralysis: quantitative evidence that EEG frequencies above 20Hz are contaminated by EMG. Clin Neurophysiol.

[27] Viertio-Oja H (2004). Description of the entropy algorithm as applied in the Datex-Ohmeda S/5 entropy module. Acta Anaesthesiol Scand.

[28] Schuller P (2015). Response of bispectral index to neuromuscular block in awake volunteers. Br J Anaesth.

[29] Bonhomme V, Hans P (2007). Muscle relaxation and depth of anaesthesia: where is the missing link?.

[30] Messner M (2003). The bispectral index declines during neuromuscular block in fully awake persons. Anesth Analg.

[31] Jordan D (2010). A program for computing the prediction probability and the related receiver operating characteristic graph. Anesth Analg.

[32] Bruhn J, Röpcke H, Hoeft A (2000). Approximate entropy as an electroencephalographic measure of anesthetic drug effect during desflurane anesthesia. Anesthesiology.

[33] Schneider G (2005). Detection of consciousness by electroencephalogram and auditory evoked potentials. Anesthesiology.

[34] Hentschke H, Stuttgen MC (2011). Computation of measures of effect size for neuroscience data sets. Eur J Neurosci.

[35] Botta-Dukát Z (2023). Quartile coefficient of variation is more robust than CV for traits calculated as a ratio. Sci Rep.

[36] Szendro P, Vincze G, Szasz A (2001). Pink-noise behaviour of biosystems. Eur Biophys J.

[37] Burch NR (1964). Period analysis of the electroencephalogram on a general-purpose digital computer. Ann NY Acad Sci.

[38] Schneider G (2014). Monitoring depth of anesthesia utilizing a combination of electroencephalographic and standard measures. Anesthesiology.

[39] Whitham EM (2007). Scalp electrical recording during paralysis: quantitative evidence that EEG frequencies above 20 Hz are contaminated by EMG. Clin Neurophysiol.

[40] Archibald JE, Drazkowski JF (1985). Clinical applications of compressed spectral analysis (CSA) in OR/ICU settings. Am J EEG Technol.

[41] Weyer C (2021). The Strength of Alpha Oscillations in the Electroencephalogram Differently Affects Algorithms Used for Anesthesia Monitoring. Anesth Analg.

[42] Kreuzer M (2020). Sleep/wake behavior and EEG signatures of the TgF344-AD rat model at the prodromal stage. Int J Mol Sci.

[43] Nardone, P., *Entropy of Difference.* arXiv:1411.0506, 2014: p. 10.

[44] Deng B (2017). Multivariate multi-scale weighted permutation entropy analysis of EEG complexity for Alzheimer's disease. Cogn Neurodyn.

[45] Popov A, Avilov O, Kanaykin O, "Permutation entropy of EEG signals for different sampling rate and time lag combinations" (2013). Signal Processing Symposium (SPS). Serock, Poland.

[46] Jing H, Takigawa M (2000). Low sampling rate induces high correlation dimension on electroencephalograms from healthy subjects. Psychiatry Clin Neurosci.

