# Supplementary material for: An in-depth analysis of parameter settings and probability distributions of specific ordinal patterns in the Shannon permutation entropy during different states of consciousness in humans
Source: J Clin Monit Comput. 2023 Jul 29;38(2):385–97. doi: 10.1007/s10877-023-01051-z (PMC10995010; doi:10.1007/s10877-023-01051-z)
Supplement: Supplementary file 1 — Supplementary file1 (DOCX 4038 kb) [file 10877_2023_1051_MOESM1_ESM.docx]

Supplemental information to: *An in-depth analysis of parameter settings and probability distributions of specific ordinal patterns in the Shannon Permutation Entropy during different states of consciousness in humans*


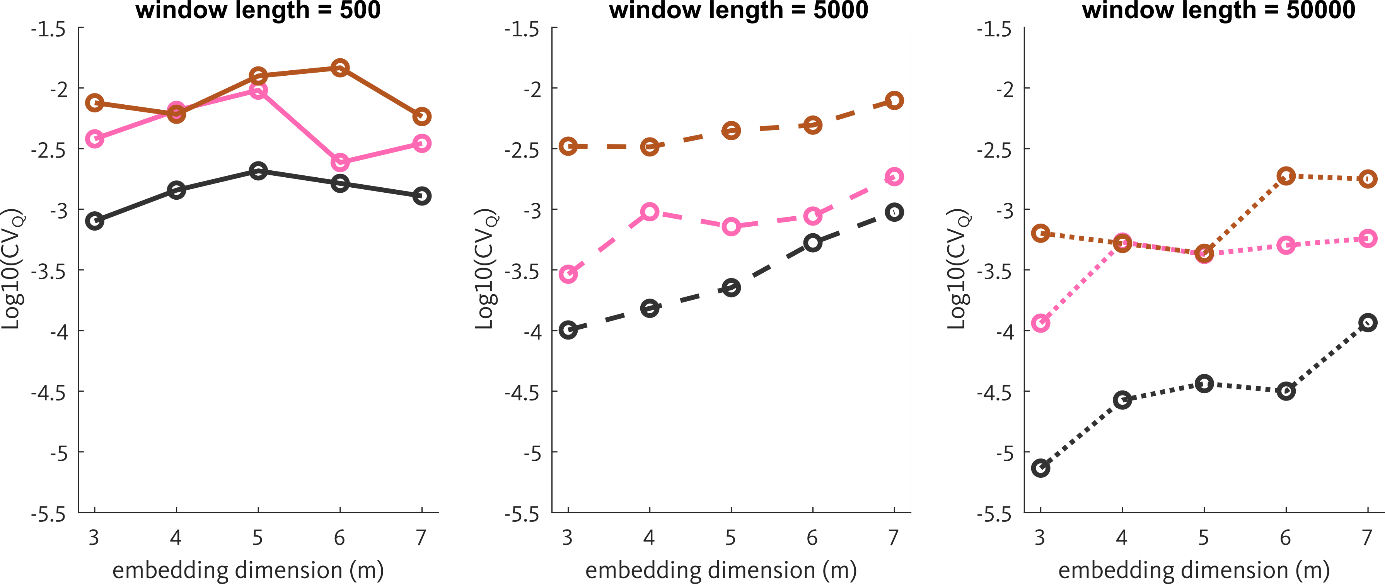


Figure S1: Quartile coefficient of variation (CVQ), fort different types of noise (white, pink, and brown) and different segment lengths.


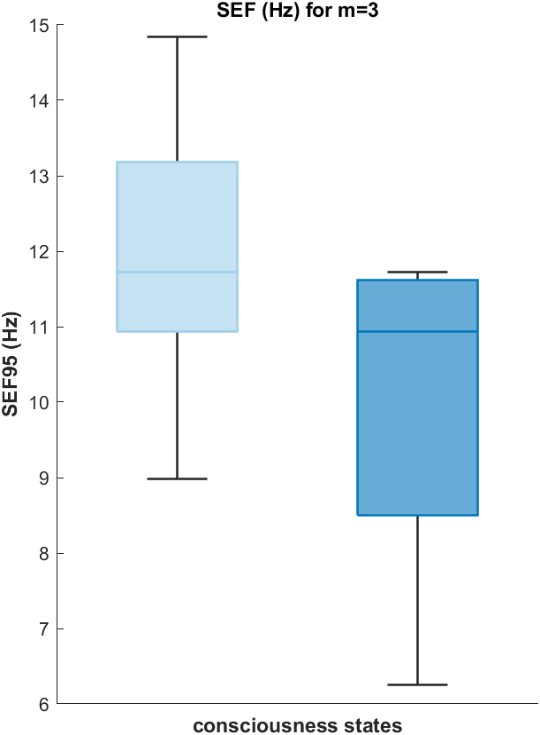


Figure S2: Spectral Edge Frequency 95 (SEF95) of lighter (inter1) and deeper (inter2) anesthestic levels.


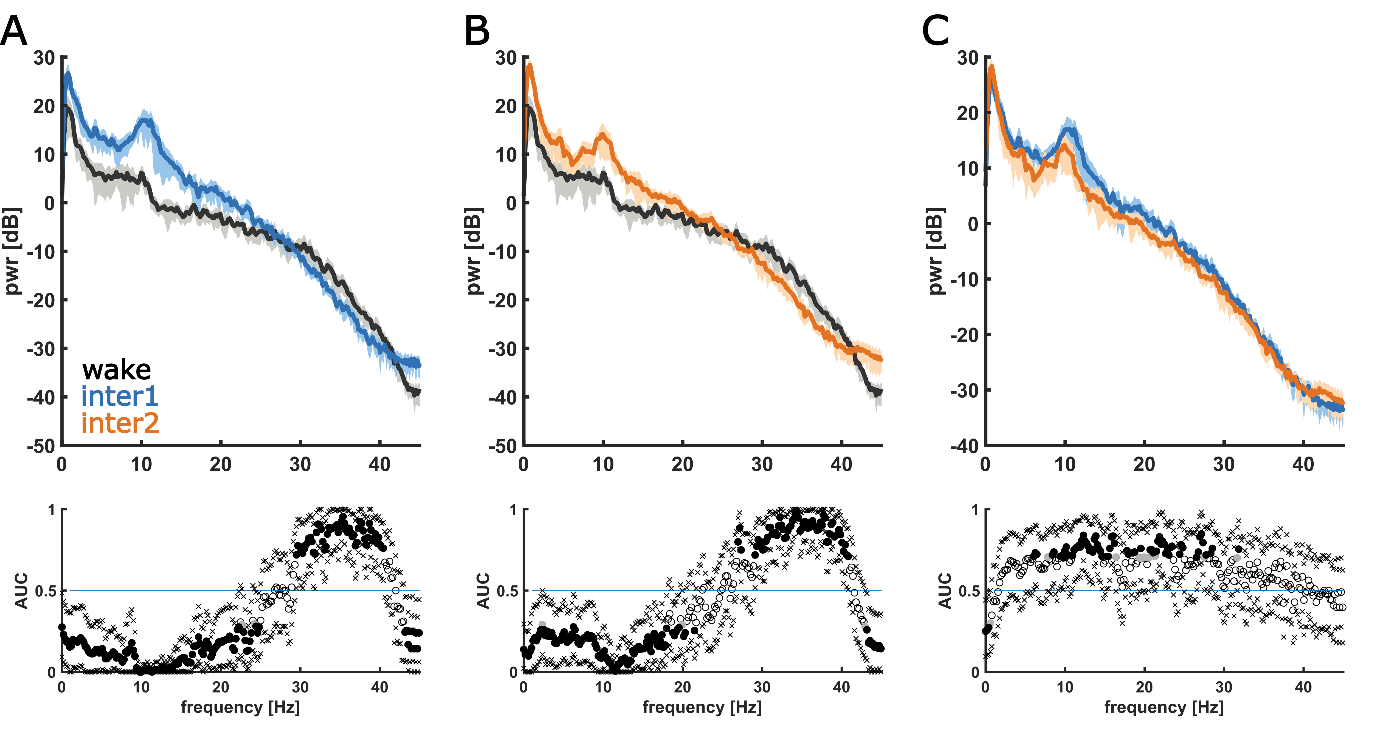


Figure S3: Group-level power spectral density plots (median and median absolute deviation) and the statistical comparison of the different anesthetic levels

1. Wake vs. inter1: The wake EEG has lower power in the slow frequencies and higher power in the higher frequencies
2. Wake vs. inter2: The wake EEG has lower power in the slow frequencies and higher power in the higher frequencies
3. Inter1 vs inter2: The EEG recorded during inter1 showed significantly higher power in the alpha-band and beta-band frequencies. Especially the more pronounced alpha-peak caused the PeEn to be lower during inter1.

The lower plots indicate the AUC values with bootstrapped 95% confidence intervals. Black dots indicate a significant difference between the groups


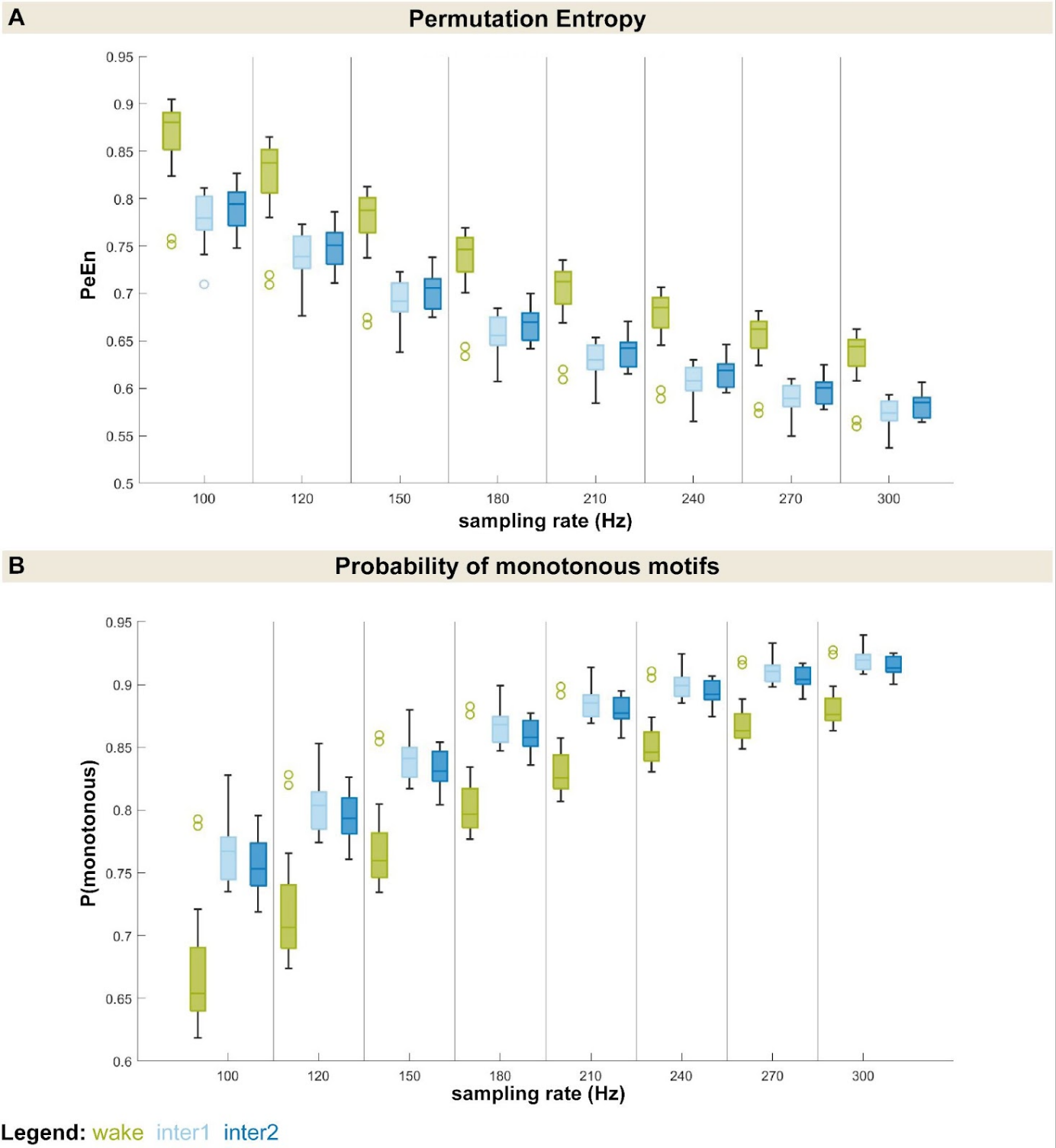

Figure S4: PeEn values for all conditions in different sample rates (100, 120, 150, 180, 210, 240, 270, 300). The separation between the conditions within one sample rate remains similar. As sample rate increases, PeEn values overall decrease for all conditions equally. Moreover, variability within the different conditions decreases as sample rate increases (4A). Figure 4B shows the Probability of monotonous motifs in increasing sampling rates. The higher the sampling rate, the higher the probability of monotonous motifs. Again, variability within the different conditions decreases as sample rate increases (4B).

Table S1: AUC values comparing the different states for varying sampling rates. Distinguishing capacity is high in wake vs. inter 1 in all sampling rates. Inter1 vs inter2 could not be distinguished, regardless of the sampling rates. Overall the AUC values did not change with sample rate


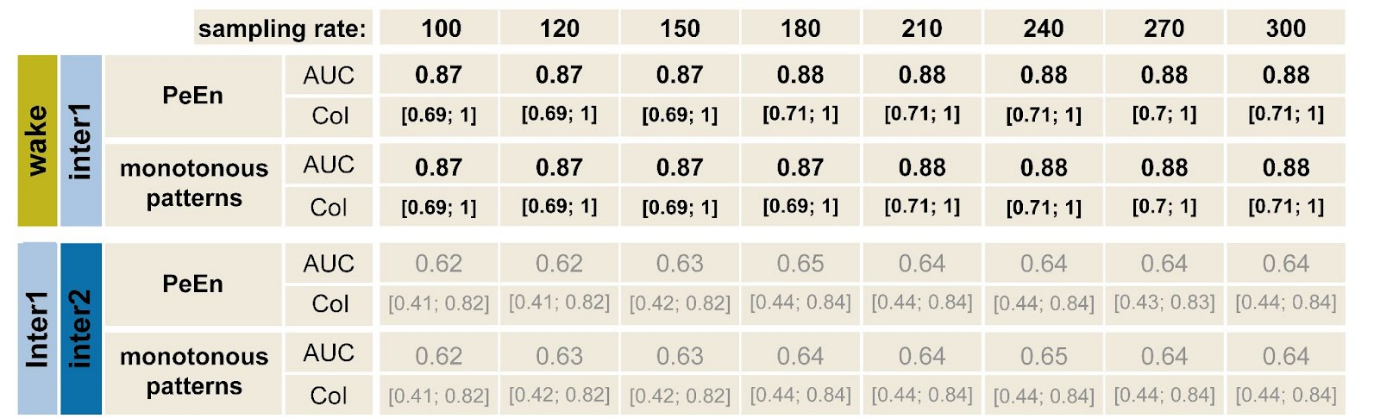


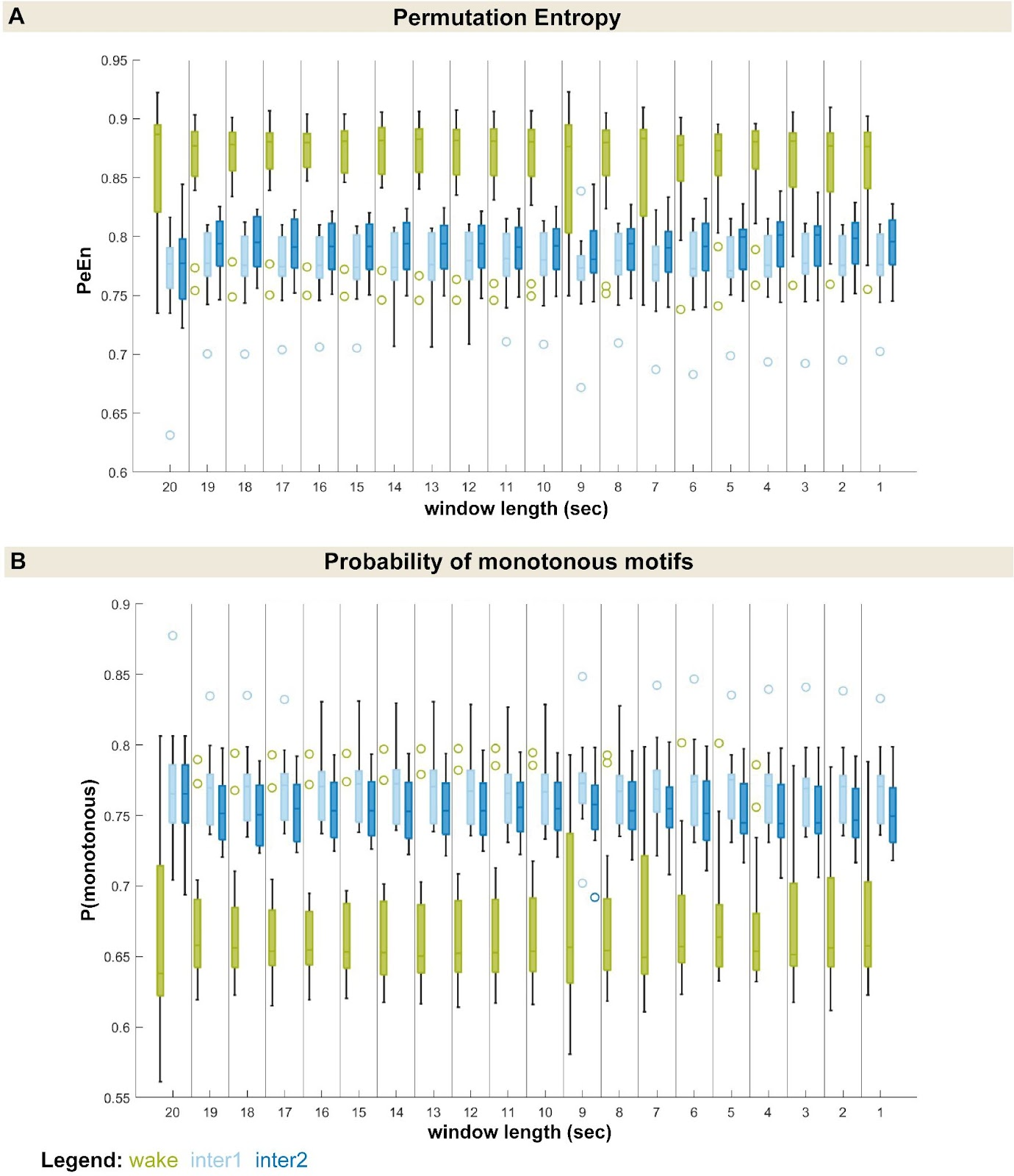


Figure S5: PeEn values for all conditions in short window lengths (<20 sec). PeEn values as well as variability within the signals stayed consistent as the signal shortens (A). The probability of monotonous patterns of all three conditions stayed consistent throughout all window lengths below 20 seconds as well (B).

Table S2: AUC values for window lengths shorter than 20sec.


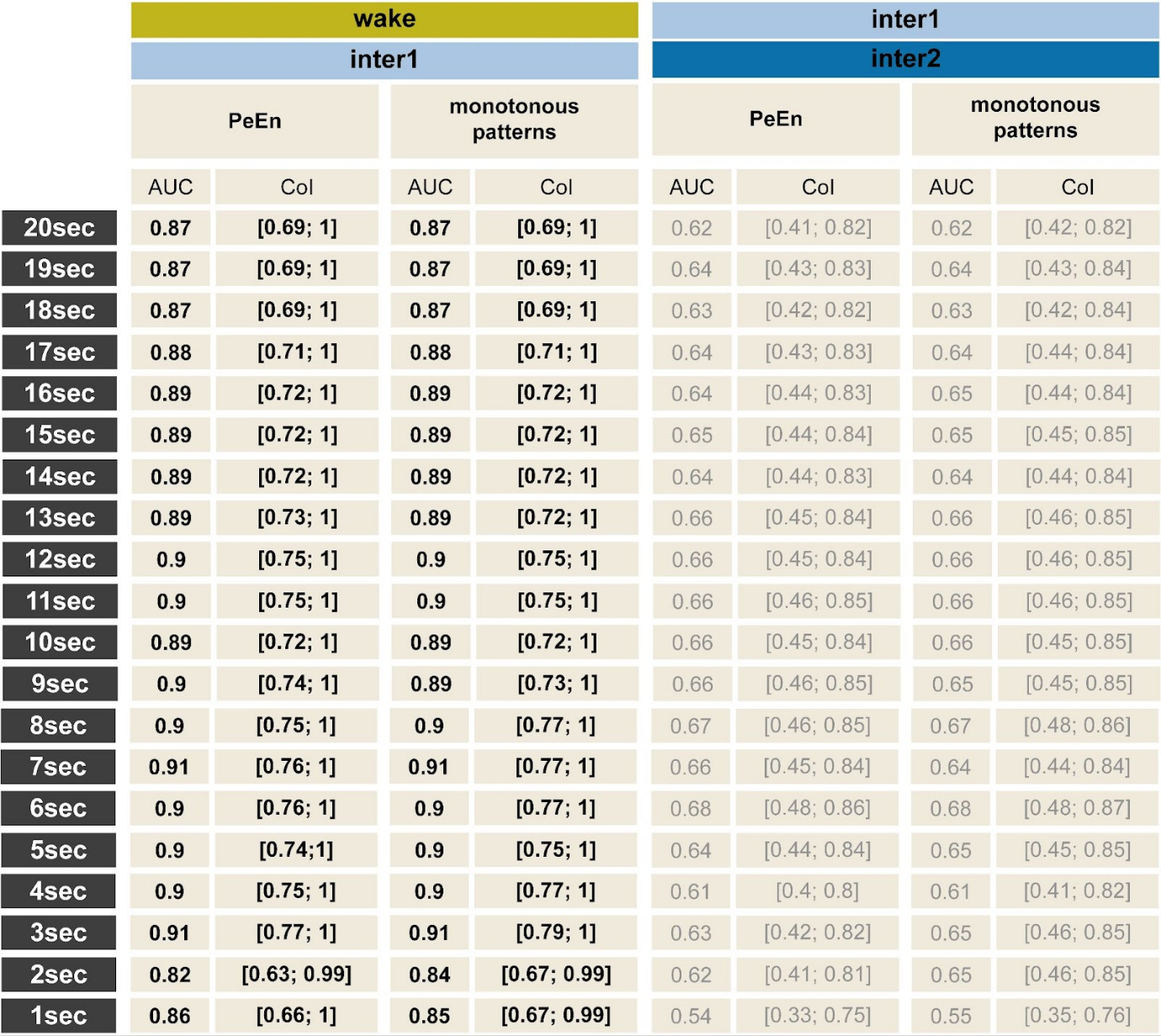


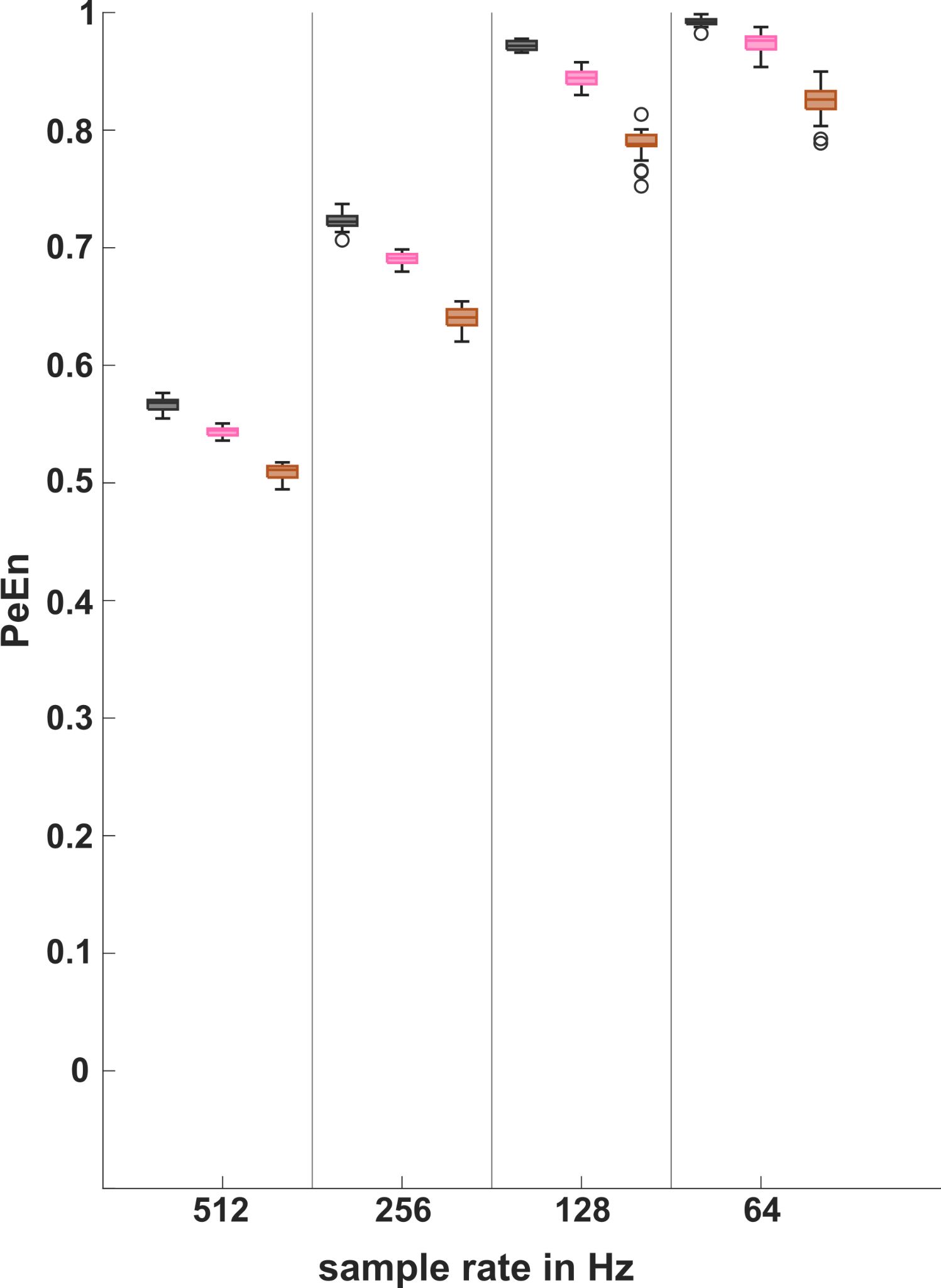


Figure S6: Box plots of the PeEn calculated for different types of noise (white noise in gray boxes, pink noise, and brown noise) and different sample rates as indicated on the y-axis. Before down-sampling a 0.5 Hz high pass and a 30 Hz low pass filter were applied.

Table S3: AUC values of the noise comparisons for different sample rates

| **Sampling Rate [Hz]** | **AUC: White vs. Pink** | **AUC: Pink vs. Brown** |
| --- | --- | --- |
| **512** | **1 [1; 1]** | **1 [1; 1]** |
| **256** | **1 [1; 1]** | **1 [1; 1]** |
| **128** | **1 [1; 1]** | **1 [1; 1]** |
| **64** | **0.99 [0.96; 1]** | **1 [1; 1]** |
